# Monitoring Viscosity and Total Solids Content of Milk Protein Concentrate Using an Inline Acoustic Flowmeter at Laboratory Scale

**DOI:** 10.3390/foods9091310

**Published:** 2020-09-17

**Authors:** Archana Bista, John T. Tobin, Colm P. O’Donnell, Norah O’Shea

**Affiliations:** 1 Food Chemistry and Technology Department, Teagasc Food Research Centre, Moorepark, P61 C996 Fermoy, Ireland; archana.bista@teagasc.ie (A.B.); john.tobin@teagasc.ie (J.T.T.); norah.oshea@teagasc.ie (N.O.); 2 School of Biosystems and Food Engineering, University College Dublin, Belfield, D4 Dublin, Ireland

**Keywords:** inline, acoustic flowmeter, viscosity, total solids, milk protein concentrate

## Abstract

Control of milk concentrate viscosity and total solids (TS) content prior to spray drying can improve dairy ingredient manufacture. However, the availability of hygienic and appropriately pressure rated process viscometers for inline monitoring of viscosity is limited. An acoustic flowmeter (FLOWave) is an inline process analytical technology (PAT) tool that measures changes in acoustic signals in response to changes in liquid properties (i.e., acoustic transmission (AT), acoustic impedance (AI), temperature and volume flowrate). In this study, an acoustic flowmeter is evaluated as an inline PAT tool for monitoring viscosity of milk protein concentrate (MPC85), protein and TS content of (MPC85), and standardised MPC (sMPC). Laboratory scale experiments were carried out at 45 °C for five different concentrations (4–21%) of MPC85 and sMPC. Results showed that AT decreased with an increase in MPC85 viscosity (e.g., AT was 98.79 ± 0.04% and 86.65 ± 0.17% for 4% and 21% TS content, respectively). Non-linear regression was carried out to develop a relationship between AT and offline viscosity (R^2^ (coefficient of determination) value = 0.97 and standard error of prediction = 1.86 mPa·s). AI was observed to increase at higher protein and TS content which was dependent on protein to total solid ratio (P_TSR). Multiple linear regression was carried out to develop the relationship between AI, protein content, TS content and P_TSR. Results demonstrated that AI could be used to monitor the protein and TS content of milk protein concentrate (R^2^ > 0.96). Overall this study demonstrated the potential of an inline acoustic flowmeter for monitoring process viscosity, protein and TS during dairy concentrate processing.

## 1. Introduction

The milk protein concentrate (MPC) powder manufacturing process involves a combination of various processes, such as membrane separation techniques, through which non-protein constituents (e.g., lactose and minerals) are extensively removed from skim milk followed by evaporation and spray drying [1]. MPC powder is used in a wide range of food applications, i.e., dairy beverages [2], cheese [3] and high protein nutritional bars [4]. The rate-limiting effect of viscosity associated with a high protein content of liquid MPC, reduces the evaporative capacity to ~30% total solids (TS) for MPC70, compared to skim milk concentrate (SMC) which can be concentrated to >50% TS prior to spray drying [5]. The viscosity of concentrate prior to spray drying affects droplet size and the rate of drying, hence monitoring and control of viscosity plays an important role in ensuring a powder of consistent quality is produced [6,7].

Many laboratory viscometers currently available have limitations, i.e., measurements are time-consuming, not suitable for rapid real-time monitoring of concentrate viscosity and may not be suitable for characterising samples with complex rheological properties (e.g., materials which are time, temperature and shear dependent) [8,9]. Some limitations of conventional viscometers can be overcome by inline viscosity measurements that monitors concentrate viscosity in real-time for improved process control [10]. Studies on the application of inline/online viscosity PAT tools in dairy processing have previously been reported [11,12,13,14,15]. O’Sullivan et al. [16] calculated inline viscosity during the reconstitution of milk powders (skim milk powder and milk protein isolate) using the Hagen-Poiseuille equation to achieve optimal powder reconstitution. Recently, Bista et al. [17] reported a strong correlation (*r* = 0.99) between viscosity measurements (measured using an inline viscometer and measured offline using a rotational rheometer) of SMC dispersion up to 40% TS. They demonstrated the potential of an inline Coriolis flowmeter for rapid and accurate monitoring of viscosity during the processing of dairy streams.

Acoustic wave sensors are low-cost PAT tools which are highly sensitive, have a fast response time and can be used to monitor the physical properties of fluid, e.g., viscosity [18]. The performance of an acoustic sensor depends on various factors, i.e., temperature, frequency change and damping of the signal in the liquid phase [19].

**Figure 1:**
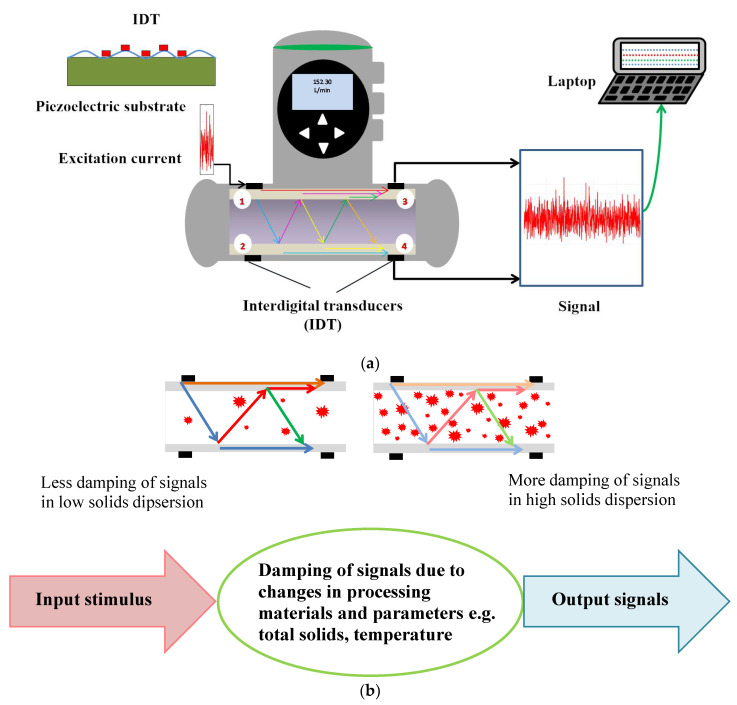
(**a**) Schematic representation and working principle of the acoustic flowmeter (FLOWave) (numbers represent four interdigital transducers). (**b**) Schematic of signals damping in low solids and high solids dispersion, arrows represent the direction of the travelling acoustic signals within the FLOwave acoustic flowmeter. The intensity of the colour of the arrow decreases as the solids content increases indicating dampening of the signals occurring.

A schematic of the operating principle of a flowmeter which uses acoustic signals in the form of a surface acoustic wave (SAW) for monitoring liquid properties is shown in Figure 1a,b. The wave starts from an initial point of excitation generated by an electric signal that generates the SAW, as shown in Figure 1a. The wave propagates along the surface of the pipe while also de-coupling into the liquid at specific angles depending on the liquid type. The receiver of the wave is an interdigital transducer (IDT) deposited on the surface of a piezoelectric substrate (e.g., quartz, lithium tantalite) and acts as both a transmitter and receiver. In this flowmeter, there are four IDT sensors on the outside of the measuring tube. The wave is detected by an IDT, which creates another wave in the opposite direction. Therefore, multiple waves are propagated along the pipe wall and into the process media, which are then detected by the opposite IDT. The time taken by the wave to travel from the sender to the receiver is dependent on the diameter of the tube and the properties of the fluid being monitored. As the surface acoustic wave propagates along the surface of the material, the velocity or amplitude of the wave is affected by changes in the propagation path [20]. The operating frequencies of surface acoustic waves are between 50 to 500 MHz [21].

The flowmeter, shown in Figure 1a, was selected for use in this study as it is low cost compared to other commercially available viscometers and gives a continuous reading during processing. It has a sanitary design with no moving parts, and the product being monitored does not come in direct contact with the sensor [22]. To the best of the authors’ knowledge, the use of an acoustic flowmeter to monitor viscosity, protein and TS content of MPC has not previously been reported. Monitoring viscosity, protein and TS content using a single instrument would facilitate improved control of the drying process and help to ensure that concentrate of an optimal TS content is pumped to the spray drier during the manufacture of dairy powders. The objectives of this study were to:

1. Evaluate the potential of an inline acoustic flowmeter (FLOWave) to monitor viscosity, protein and TS content of reconstituted MPC85 of varying TS content (4–21%) at laboratory scale.
2. Develop mathematical models using inline acoustic parameters to predict viscosity, protein and TS content in concentrated dairy systems.

## 2. Materials and Methods

### 2.1. Raw Materials

Milk protein concentrate (MPC85) powder and lactose powder both were supplied by Glanbia Ingredients (Cavan, Virginia, Ireland). The MPC85 powder had a moisture content of 3.98%, protein content of 86.08%, fat content of 1.39%, lactose content of 0.71% and ash of 6.59%.

### 2.2. Experimental Laboratory Set-Up

#### 2.2.1. Sample Preparation—MPC85

MPC85 was rehydrated to −22% (*w*/*w*) TS at 50 °C with reverse osmosis (RO) water using a high shear mixer (YTRON-Z, 1.50FC, YTRON Process Technology GmbH, Bad Endorf, Germany). Mixing was performed for 5 min to facilitate complete dissolution of the powder in water. The concentrate was chilled at 4 °C overnight and used within 24 h. Prior to analysis, the concentrate was diluted with RO water to the desired TS content, i.e., 4%, 10%, 12%, 16% and 21% (*w*/*w*) TS. Additional concentrates i.e., 7% and 21% (*w*/*w*) TS were used as validation points. The TS content of each reconstituted sample was confirmed using a microwave moisture analyser (Smart trac, 5 turbo, CEM Corporation, Matthews, NC, USA) prior to flow experiments.

#### 2.2.2. Sample Preparation—sMPC

**Figure 2:**
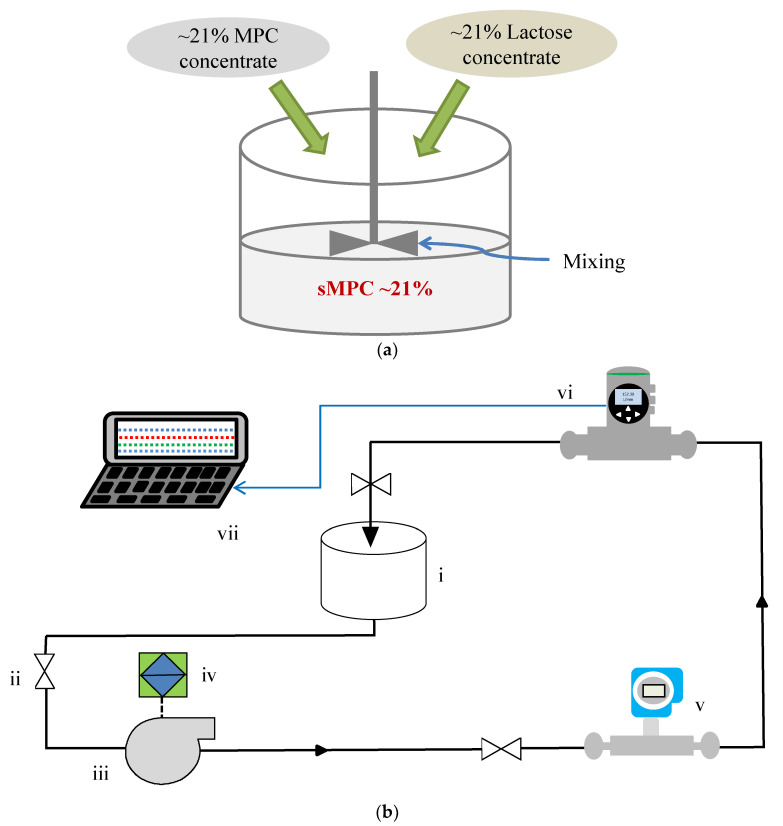
(**a**) Preparation of standardised milk protein concentrate (sMPC) with addition of lactose concentrate. (**b**) Schematic of an inline acoustic flowmeter in a laboratory scale skid. (i) Five litre tank; (ii) globe valve; (iii) centrifugal pump; (iv) variable speed drive; (v) inline Coriolis flowmeter; (vi) inline acoustic flowmeter; (vii) laptop.

sMPC (i.e., MPC85 with reduced protein) was prepared with the addition of rehydrated lactose concentrate to rehydrated MPC85 of varying TS content, to evaluate the potential of the FLOWave acoustic flowmeter to detect compositional changes. Lactose was rehydrated to ~21% (*w*/*w*) TS at 85 °C using RO water and mixing was carried out for 20 min after the addition of the lactose powder. MPC85 and lactose concentrate of a similar TS content were mixed together, e.g., 5 kg of 21% MPC85 concentrate and 5 kg of 21% lactose concentrate to obtain 10 kg of 21% sMPC (Figure 2a). sMPC concentrate samples were prepared with TS content as outlined for reconstituted MPC85, i.e., 4% to 21% TS. Prior to commencing experiments, the TS content of all sMPC samples was determined using a microwave moisture analyser (Smart trac, 5 turbo, CEM Corporation, Matthews, NC, USA).

#### 2.2.3. Experimental Laboratory Scale Flow Set-Up—MPC85 and sMPC

A laboratory-scale skid previously described by Bista et al. [17] was used to evaluate the inline acoustic flowmeter (FLOWave, Bürkert, Germany) to monitor the viscosity of reconstituted MPC85, protein and TS content of reconstituted MPC85 and sMPC (Figure 2b). A centrifugal pump (GEA, Düsseldorf, Germany) was used to circulate reconstituted MPC85 and sMPC in a continuous loop. Based on previous preliminary trials, the frequency of the variable speed drive that controlled the pump was set to 20 Hz for all experiments to achieve stable reading at the highest TS content (21% MPC85). All experiments were carried out at 45 °C (the typical processing temperature of MPC concentrate post evaporation and prior to spray drying), and this temperature was maintained using a circulating water bath (Grant TX150, Grant Instruments, Cambridgeshire, UK) connected to a tank with a heating coil. The flowrates for 4% to 21% TS in this experiment ranged from 6.63 ± 0.33 to 5.21 ± 0.22 l/min for MPC85 and 7.51 ± 0.23 to 7.20 ± 0.32 l/min for sMPC, respectively. The effect of flowrate on the measured viscosity of MPC85 was tested at the highest TS (i.e., 21% TS) and experiments were carried out at 45 °C at three different flowrates (3, 5 and 6.3 l/min). These flowrates were maintained using a back pressure valve. The inline viscosity at the three different flowrates in the skid was monitored using a Coriolis flowmeter (Proline Promass I300, Endress + Hauser, Switzerland) and an acoustic flowmeter (FLOWave, Bürkert, Germany). The approximate shear rate in the pipe was calculated using the following equation [16].

(1) $$\mathrm{Shear}\;\mathrm{rate}\;\left(s^{-1}\right)=\frac{8v}{d},\;where,v=\frac{Q}{A}$$

where *v* is the average velocity (m·s^−^^1^) of the concentrate, *d* is the diameter of the pipe (17 mm), *Q* is the volumetric flowrate (m^3^·s^−^^1^), and *A* is the cross-sectional (m^2^) area of the pipe.

Data generated from the acoustic flowmeter was collected using a “Bus-stick Communicator” (Bürkert, Germany), and the recorded data was monitored using “Bürkert-Communicator” software (Bürkert, Germany). Once acoustic transmission (AT) and acoustic impedance (AI) were stable at the desired temperature, acoustic data and temperature were collected at 10 s intervals for 10 min for each concentrate sample. All experiments were performed in triplicate.

### 2.3. MPC85 and sMPC Measurements

#### 2.3.1. Reference Apparent Viscosity Measurements

Apparent viscosity measurements of MPC85 samples were performed at a shear rate of 300 s^−^^1^ and a temperature of 45 °C using a rotational rheometer (MCR 302, Anton Paar GmbH, Austria) with a concentric cylinder system (CC27, Anton Paar, GmbH, Austria). As viscosity is time, temperature, shear and solid content dependent, only one component can be varied at a time. In this study temperature and shear rate were kept constant, and TS content was varied. Therefore, a reference shear rate of 300 s^−^^1^ was selected after performing a shear ramp (0 s^−1^ to 500 s^−1^ at a temperature of 45 °C) as it is important to determine the apparent viscosity in a Newtonian region to determine if the flowmeter was sensitive to changes in viscosity arising from increases in TS content independently. The shear rate versus shear stress curves was evaluated using a power law relationship to obtain consistency coefficient (k) and flow behaviour index (n) as described by Reference [23]. If n = 1, the sample is Newtonian, whereas n < 1 and n > 1 indicates shear thinning and shear thickening behaviour. All the experiments were performed in triplicate, and two repeat measurements were performed per replicate.

#### 2.3.2. Protein Content

The protein content of MPC85 and sMPC samples were determined using the Kjeldahl method [24]. Two repeat measurements were performed per replicate.

#### 2.3.3. Density

The density of MPC and sMPC were determined using a portable density and concentration meter (DMA^TM^ 35, Anton Paar, Graz, Austria) at 43 °C. Two repeat measurements were performed per replicate.

### 2.4. Statistical Methods

The results are presented as the mean ± standard deviation of three replicates. To obtain a correlation between data collected from an inline acoustic flowmeter and offline rotational rheometer a Spearman and Pearson correlation was performed using SigmaPlot 14.0 and regression analysis was performed using Minitab (version 17.1.0, Lead Technologies, Inc., State College, PA, USA). Non-linear regression was performed on the experimental data to develop prediction equations, and statistical evaluation of equations were carried out using the coefficient of determination (R^2^), standard error of prediction (SEP), error sum of squares (SSE), root mean square error (RMSE), Bayesian information criterion (BIC) and Akaike information criterion (AIC) to predict the relationship between apparent viscosity (mPa·s) and AT [25]. Multiple linear regressions were performed on the experimental data to develop prediction equations between AI, protein and TS content. The goodness of fit between experimental data (measured using instrument) versus predicted values (predicted using the regression equation) was evaluated using R^2^ and SEP. Analysis of variance (ANOVA) was used to determine statistical significance. *p* < 0.05 was considered as statistically significant.

## 3. Results and Discussion

### 3.1. Effect of Total Solids Content on Apparent Viscosity of MPC85

Apparent viscosity ranged from 2.30 ± 0.03 to 28.55 ± 2.95 mPa·s for 4% and 21% TS samples, respectively. The flow behaviour index value ranged from 1 ± 0.071 to 0.61 ± 0.058, and consistency coefficient ranged from 0.001 to 0.51 Pa s^n^ for 4% to 21% TS samples, respectively. Analysis of the apparent viscosity profile using the power law, highlighted the differences in the flow behaviour. The flow behaviour index 1 at 4% TS demonstrated the Newtonian behaviour, and the value decreased to 0.62% at 21% TS indicating that as the protein content increased, the samples became more shear thinning.

**Figure 3:**
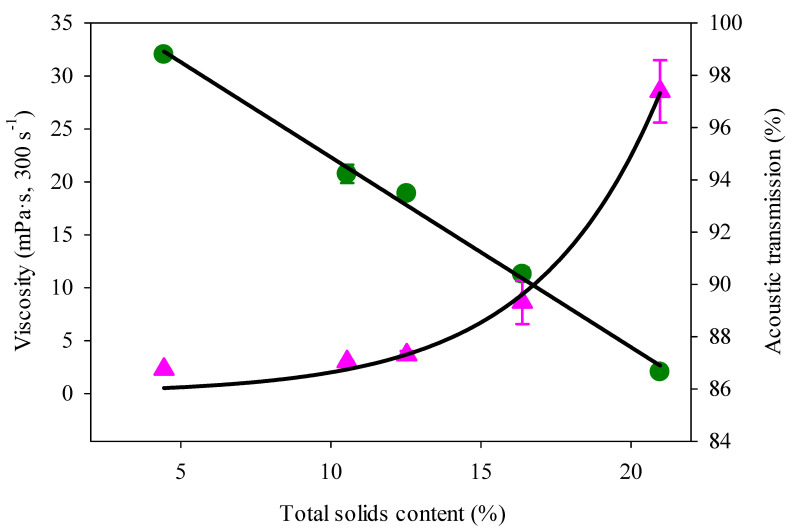
Apparent viscosity (▲) and acoustic transmission (●) of MPC85 as a function of total solids (TS) content (4–21%) at 45 °C.

The apparent viscosity of MPC85 concentrate increased linearly up to 12% TS with an exponential increase at higher TS content, as shown in Figure 3. The linear increase in apparent viscosity at lower TS is due to the greater inter-particle distance, which allows more space for particles to move freely, resulting in a lower apparent viscosity. In comparison, the exponential increase in apparent viscosity observed at higher TS content can be attributed to the higher protein content, which increased at higher TS [26]. The viscosity of dairy concentrates (e.g., skim milk concentrate) increases with protein content, due to an increase in the volume fraction occupied by the additional amount of protein particles present [27]. At a higher protein content, the inter-particle distance between micelles becomes considerably smaller, due to an increased micelle-micelle interaction, thus resulting in a higher volume fraction of casein micelles [7]. Dispersions with a high casein content (180 g/L) cannot move freely, due to casein not having enough space to move. This phenomenon results in a high viscosity at high protein content and hence would explain the high apparent viscosity values observed at higher TS content of MPC85 (18% protein at 21% TS) in this study. A similar increase in viscosity of reconstituted MPC80 (19%, 21% and 23% TS) was observed by Rupp, i.e., Reference [28] and by O’Donnell and Butler [29] for MPC (20–26% TS) samples. Bista et al. [17] observed an exponential increase in viscosity as a function of TS content of reconstituted SMC (10–40% TS). Li et al. [30] also reported an increase in viscosity that corresponds to an increase in protein content (8–12.8%) in protein and polysaccharide emulsions of varying ratios (1:1–4:1). The authors proposed that at a higher protein content, protein molecules swell and move closer together—resulting in a higher viscosity.

#### 3.1.1. Evaluation of an Inline Acoustic Flowmeter to Monitor MPC85 Viscosity at Different Total Solids Content

Acoustic transmission (AT) of MPC concentrates was monitored using an inline acoustic flowmeter. AT (%) was determined from the amplitude changes of different parameter signals travelling through samples. The AT of water without gas bubbles at 20 °C corresponds to an AT of 100%. AT of MPC85 concentrate was highest at 4% TS (98.79 ± 0.04%) and lowest at 21% TS (86.65 ± 0.17%). At a low TS content, the number of particles present in the MPC concentrate is lower, and the dampening effect of the particles on the signals is minimal. Hence the AT signals can easily reach the IDTs resulting in a higher AT value being observed. The AT of MPC85 decreased linearly with an increase in TS content (4–21%), as shown in Figure 3. With an increase in TS content, the number of particles in the concentrate increases and the signal reaching the IDTs is dampened by the additional particle present in the concentrate (Figure 1b) and hence the AT value decreases with increasing TS content.

**Figure 4:**
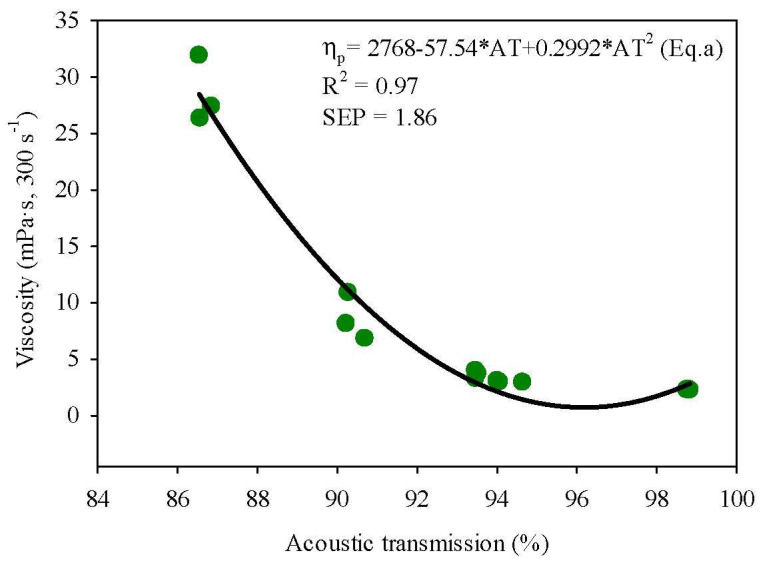
Apparent viscosity (mPa·s) of MPC85 of 4–21% TS measured using the reference method (rotational rheometer) versus AT (%) measured using an inline acoustic flowmeter. η_p_, viscosity of MPC85 predicted by prediction Equation (a); AT, acoustic transmission measured using an inline acoustic flowmeter; R^2^, coefficient of determination; SEP, standard error of prediction.

A direct comparison of AT values with apparent viscosity values is not feasible, due to the differences in the working principle of the instruments. However, a relationship between the values measured from the instruments can be determined [31]. AT data of MPC85 measured using an inline acoustic flowmeter correlated negatively (*r* = −0.985, *p* < 0.05) with apparent viscosity measured using an offline rotational rheometer as illustrated in Figure 4. Table 1 shows the prediction equations along with statistical parameters employed to evaluate the models. The R^2^ value of 0.97 suggests that the polynomial prediction model Equation (a) can be used to describe the relationship between apparent viscosity and AT compared to other equations tested.

MPC85 at 7% and 19% TS were not included in the data points of prediction Equation (a), as these data points were used to validate the robustness of prediction Equation (a). The measured apparent viscosity values for the validation points were obtained using the reference method, and the predicted values were derived from the prediction Equation (a). As can be seen from Supplementary Figure S1, the predicted values and measured values were highly correlated. The measured reference values and predicted values generated from the prediction Equation (a) were fitted to a linear regression model to evaluate the performance of the prediction Equation (a). An R^2^ value of 0.97 with a SEP 1.76 mPa·s was obtained demonstrating that the inline acoustic flowmeter investigated can predict the apparent viscosity of MPC85 under similar operating conditions to industry, i.e., TS content, temperature and flowrates.

The apparent viscosity of sMPC for all concentrate samples was lower than MPC85, due to the addition of lactose, i.e., (<4 mPa·s), hence apparent viscosity of sMPC is not presented in this study.

#### 3.1.2. Effect of Flowrate and Shear Rate on MPC85 Viscosity and Acoustic Transmission at 21% TS

The effect of flowrate in the pipe and shear rate in a rotational rheometer was studied. The inline viscosity and AT of MPC85 at 21% TS was measured at selected flowrates as described (in Section 2.2.3. No significant difference was observed (*p* < 0.05) in the viscosity and AT of 21% MPC85 at different flowrates (Supplementary Table S1). Concentrate viscosity was independent of the flowrates studied, most likely as a result of applied shearing to the concentrate from the shear force generated during centrifugal pumping. The centrifugal pump creates centrifugal force, due to rotation of impeller, thus increasing fluid velocity during processing, and applying the shearing effect on the concentrate [32].

The apparent viscosity of MPC85 at 21% TS content measured using a rotational rheometer decreased with increasing shear rate demonstrating a shear thinning behaviour up to a shear rate of ~180 s^−^^1^ over a shear ramp (0 s^−^^1^ to 600 s^−^^1^) (Supplementary Figure S2). With increasing shear rates at higher TS content, the weaker bonds (ionic and hydrogen) are disrupted by hydrodynamic forces resulting in the breakdown of the structure and lower apparent viscosity [7]. MPC85 behaved as a Newtonian fluid at shear rates above 180 s^−^^1^ (Supplementary Figure S2). The calculated shear rate in the pipe using Equation (1) for the lowest flowrate employed was 180 s^−^^1^, and for all other flowrates, the calculated shear rate was higher than 180 s^−^^1^. Therefore, the offline apparent viscosity values for all MPC85 samples was measured at a constant shear rate of 300 s^−^^1^.

### 3.2. Evaluation of an Inline Acoustic Flowmeter to Monitor Protein/Total Solids Content of MPC85

**Figure 5:**
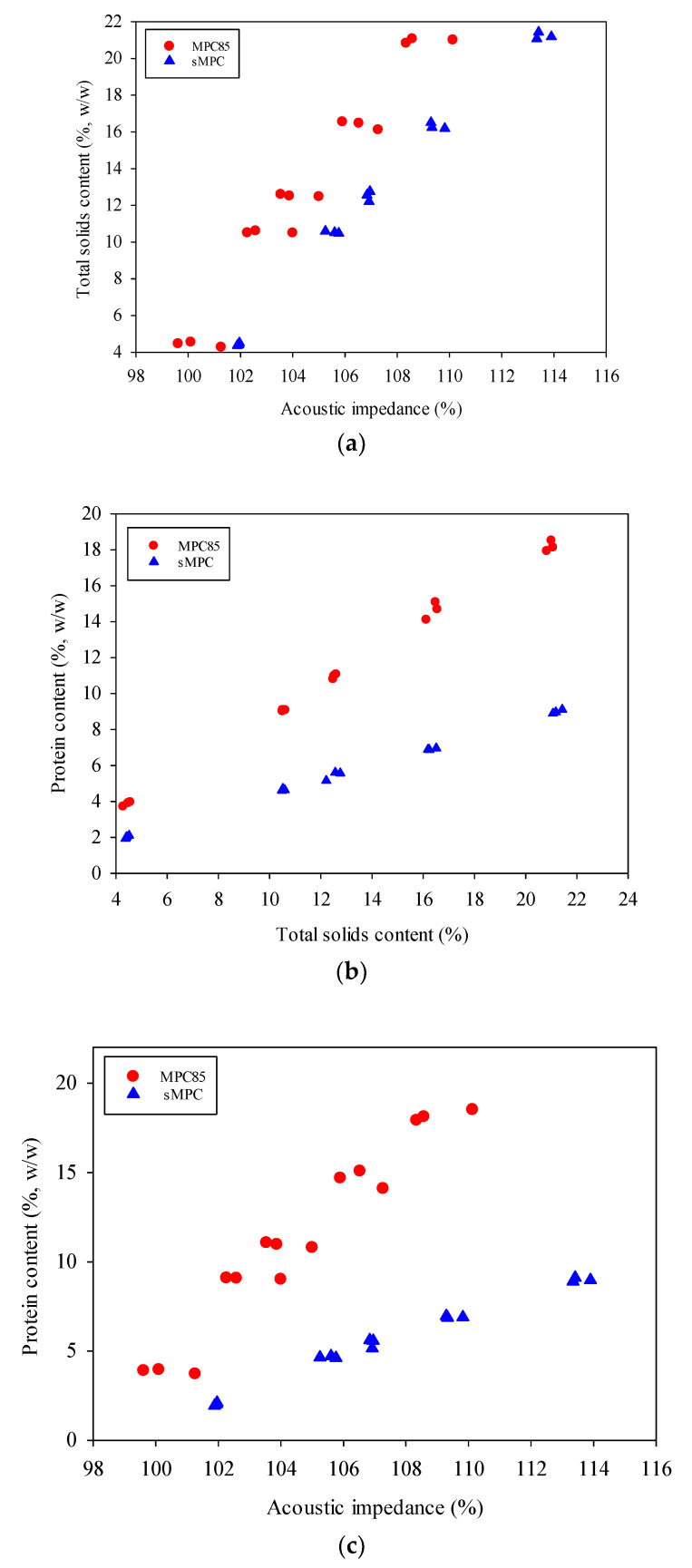
(**a**) TS content (4–21% TS) versus AI of MPC85 (●) and sMPC (▲) samples. (**b**) Protein versus TS content (4–21% TS) of MPC85 and sMPC samples. (**c**) Protein versus AI of MPC85 and sMPC samples.

The FLOWave acoustic flowmeter also measures AI of a liquid and is determined by Equation (2), where, AI is acoustic impedance, *ρ* is the density and *C* is the sound velocity [33,34]. AI has been previously used to monitor the density of non-food samples, for example, during the manufacture of liquid polypropylene and clay slurries [35,36]. Both of these studies were carried out to evaluate the potential of using AI to monitor density for process optimisation. The use of AI to monitor TS content of dairy concentrates has not been reported to date. The AI value of water is calibrated to ca. 100% at room temperature and increases with an increase in TS/protein content. AI values of MPC85 ranged from 100.32 ± 0.85% to 109.02 ± 0.97% for 4% TS and 21% TS, respectively, and increased linearly with an increase in TS content, as shown in Figure 5a. Equation (2) shows that AI is directly proportional to density which is a function of composition.

(2) AI=ρ×C

The density of the concentrate samples (4–21%) increased linearly with the increase in TS content (Supplementary Figure S3), and hence, AI also increased.

To investigate the relationship between AI signal and protein content, additional trials were carried where TS was kept similar in both MPC85 and sMPC samples. The protein content of all sMPC samples was reduced through the addition of lactose as described in Section 2.2.2. The AI values of the sMPC samples ranged from 101.93 ± 0.04% to 113.55 ± 0.31% for 4% TS and 21% TS samples, respectively, and increased linearly with increasing TS content (Figure 5a). The protein content of MPC85 and sMPC increased linearly with an increase in TS content (Figure 5b). Although the TS content was similar in both MPC85 and sMPC, the protein content was lower in sMPC, and the AI values of all sMPC samples were higher than MPC85 samples. The addition of lactose increased the density of sMPC (Supplementary Figure S3). Therefore, the higher density of the concentrate may have resulted in greater resistance against the travelling sound wave, thus increasing the AI of sMPC samples.

The above results suggest that the relationship between protein and AI depends on the protein to TS ratio (P_TSR). For example, if the AI is 109%, the protein content can be ~5% for P_TSR of 0.44 (for sMPC) or ~17% for P_TSR of 0.87 for MPC85 depending on the P_TSR (Figure 5c). Multiple linear regression (MLR) was carried out to investigate the relationship between the independent variables (protein, TS content and P_TSR) and the dependent variable (AI). The P_TSR is calculated as follows:

(3) $$P\_\mathrm{TSR}=\frac{\mathrm{Protein}\;\mathrm{content}}{\mathrm{Total}\;\mathrm{solids}\;\mathrm{content}}$$

The following prediction equations were developed to predict the protein and TS content of MPC85 and sMPC samples:

(4) $$\mathrm{Protein}\left(\%,w/w\right)=\beta_{1}+\beta_{2}\times P\_\mathrm{TSR}+\beta_{3}\times \mathrm{AI}\times P\_\mathrm{TSR}$$

where, *β*_1_ = −4.17, *β*_2_ = −159.2 and *β*_3_ = 1.70

(5) $$\mathrm{Total}\;\mathrm{solids}\left(\%,w/w\right)=\beta_{1}+\beta_{2}\times \mathrm{AI}+\beta_{3}\times \mathrm{AI}\times P\_\mathrm{TSR}$$

where, *β*_1_ = −154.63, *β*_2_ = 1.5168 and *β*_3_ = 0.0982

**Figure 6:**
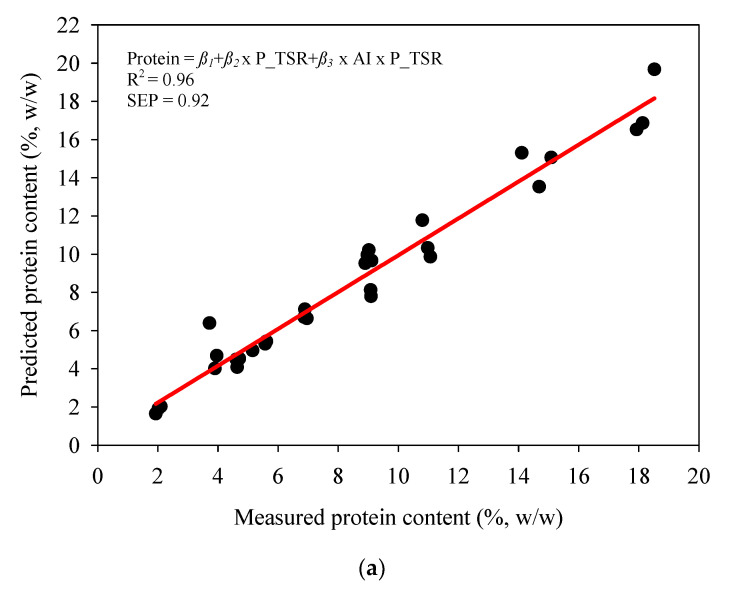

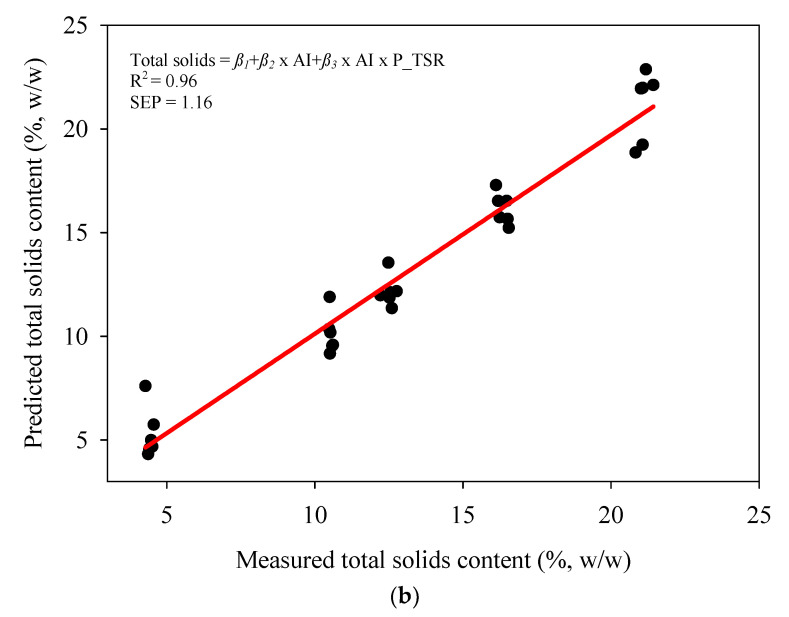
(**a**) Predicted versus measured protein content of MPC85 and sMPC samples of 4–21% TS. (**b**) Predicted versus measured total solids content of MPC85 and sMPC samples of 4–21% TS. AI, acoustic impedance measured using an inline acoustic flowmeter; P_TSR, protein to TS ratio: R^2^, coefficient of determination; SEP, standard error of prediction.

The significant parameters affecting protein content from Equation (4) based on *p*-values < 0.0005 were P_TSR and AI × P_TSR. Similarly, the significant parameters affecting TS content from Equation (5) were AI and AI × P_TSR. Plots of measured and predicted protein content and TS content along with R^2^ and SEP values are shown in Figure 6a,b. R^2^ values > 0.96 for both protein and TS prediction equations demonstrated that the developed Equations (4) and (5) are suitable to describe the relationship between AI, TS, protein content and P_TSR (i.e., if AI and P_TSR are known TS and protein content can be predicted using the developed equations). Use of these equations would be beneficial during the reconstitution of dairy powders as currently the availability of a rapid, cost-effective method to monitor reconstitution of dairy powders is limited [37]. In addition, poor control of dairy powder reconstitution can have negative impacts on the functional and nutritional properties (e.g., protein content, water holding capacity) of final products [38].

## 4. Conclusions

This study evaluated an inline acoustic flowmeter (FLOWave) as a PAT tool to monitor the viscosity of MPC85, protein and TS content of MPC85 and sMPC of varying concentration (4–21% w/w) at laboratory scale at 45 °C. Non-linear regression was performed between AT and viscosity, and multiple linear regression was performed between AI, P_TSR, protein and TS content to develop prediction equations. R^2^ values > 0.96 demonstrated the potential of the inline acoustic flowmeter for monitoring inline viscosity, protein and TS content during processing. Further studies are required at industry scale and at higher TS to validate the potential of the acoustic flowmeter evaluated for monitoring process viscosity, protein and TS content in dairy powder reconstitution processes.

## Figures and Tables

**Table 1:** Parameters and regression co-efficient of the different models applied to predict apparent viscosity using acoustic transmission (AT). R^2^, coefficient of determination; SEP, standard error of prediction; SSE, error sum of squares; RMSE, root mean square error; BIC, Bayesian information criterion; AIC, Akaike information criterion.

Model	Equation	R^2^	SEP	SSE	RMSE	BIC	AIC
Polynomial	η_p_ = 2768 − 57.54 × AT + 0.2992 × AT^2^ (a)	0.97	1.86	96.11	2.53	32.25	39.86
Exponential	η_p_ = 2 × 10^9^ × e^−0.213 × AT^	0.89	2.21	110.80	2.72	38.18	32.07
Power	η_p_ = 7 × 10^39^ × AT^−19.89^	0.90	2.03	133.96	2.99	41.02	34.92

## Supplementary Materials

The following are available online at https://www.mdpi.com/2304-8158/9/9/1310/s1, Figure S1: Linear correlation between the predicted and measured MPC85 viscosities at laboratory scale (45 °C, 300 s^−1^), Figure S2: Viscosity of MPC85 of 21% TS as a function of shear rate (0–600 s^−1^) at 45 °C, Figure S3: Density of MPC85 and sMPC concentrate at 45 °C as a function of TS content (4–21%), Table S1: Effect of flowrates on the viscosity of MPC85.

## References

[1] Lagrange V., Whitsett D., Burris C. (2015). Global Market for Dairy Proteins. J. Food Sci..

[2] Pandalaneni K., Amamcharla J.K., Marella C., Metzger L.E. (2018). Influence of milk protein concentrates with modified calcium content on enteral dairy beverage formulations: Physicochemical properties. J. Dairy Sci..

[3] Francolino S., Locci F., Ghiglietti R., Iezzi R., Mucchetti G. (2010). Use of milk protein concentrate to standardize milk composition in Italian citric Mozzarella cheese making. LWT Food Sci. Technol..

[4] Banach J.C., Clark S., Lamsal B.P. (2018). Extrusion modifies some physicochemical properties of milk protein concentrate for improved performance in high-protein nutrition bars. J. Sci. Food Agric..

[5] Agarwal S., Beausire R.L.W., Patel S., Patel H. (2015). Innovative uses of milk protein concentrates in product development. J. Food Sci..

[6] O’Callaghan D., Cunningham P. (2005). Modern process control techniques in the production of dried milk products—A review. Lait.

[7] Bienvenue A., Jimenez-Flores R., Singh H. (2003). Rheological properties of concentrated skim milk: Importance of soluble minerals in the changes in viscosity during storage. J. Dairy Sci..

[8] Lu X., Hou L., Zhang L., Tong Y., Zhao G., Cheng Z.Y. (2017). Piezoelectric-excited membrane for liquids viscosity and mass density measurement. Sens. Actuator A Phys..

[9] Trinh B., Trinh K.T., Haisman D. (2007). Effect of total solids content and temperature on the rheological behaviour of reconstituted whole milk concentrates. J. Dairy Res..

[10] Cullen P.J., Duffy A.P., O’Donnell C.P., O’Callaghan D.J. (2000). Process viscometry for the food industry. Trends Food Sci. Technol..

[11] Lin T.I., de Souza G., Young B. (2009). Towards a viscosity and density correlation for dairy fluids—A soft sensor approach. Computer Aided Chemical Engineering, Proceedings of the 10th International Symposium on Process Systems Engineering, Salvador, Brazil, 16–20 August 2009.

[12] O’Donnell C.P., Herlihy N., McKenna B.M., Yano T., Matsuno R., Nakamura K. (1994). Use of an in-line vicosmeter in the manufacture of skim milk powder 1994. Developments in Food Engineering, Proceedings of the 6th International Congress on Engineering and Food, Chiba, Japan, 23–27 May 1994.

[13] Schuck P., Mejean S., Dolivet A., Beaucher E., Famelart M.H. (2005). Pump amperage: A new method for monitoring viscosity of dairy concentrates before spray drying. Lait.

[14] O’Callaghan D., Schulz D., O’Donnell C., Duffy A., Hade J., Howard V. Improved Control in Dairy Processing. https://www.milktronics.com/wp-content/uploads/2017/07/Improved-Control-in-Dairy-Processing-2.pdf..

[15] Pu Y., O’Shea N., Hogan S.A., Tobin J.T. (2020). Assessment of a solid-state bulk acoustic wave sensor to measure viscosity of Newtonian and Non-Newtonian fluids under static and flow conditions. J. Food Eng..

[16] O’Sullivan J.J., Schmidmeier C., Drapala K.P., O’Mahony J.A., Kelly A.L. (2017). Monitoring of pilot-scale induction processes for dairy powders using inline and offline approaches. J. Food Eng..

[17] Bista A., Hogan S.A., O’Donnell C.P., Tobin J.T., O’Shea N. (2019). Evaluation and validation of an inline Coriolis flowmeter to measure dynamic viscosity during laboratory and pilot-scale food processing. Innov. Food Sci. Emerg. Technol..

[18] Raimbault V., Rebiere D., Dejous C. (2008). A microfluidic surface acoustic wave sensor platform: Application to high viscosity measurements. Mater. Sci. Eng. C.

[19] Mujahid A., Dickert L.F. (2017). Surface acoustic wave (saw) for chemical sensing applications of recognition layers. Sensors.

[20] Drafts B. (2001). Acoustic wave technology sensors. IEEE Trans. Microw. Theory Tech..

[21] Gronewold T.M.A. (2007). Surface acoustic wave sensors in the bioanalytical field: Recent trends and challenges. Anal. Chim. Acta.

[22] O’Shea N., O’Callaghan T.F., Tobin J.T. (2019). The application of process analytical technologies (PAT) to the dairy industry for real time product characterization—Process viscometry. Innov. Food Sci. Emerg. Technol..

[23] Anema S.G., Lowe E.K., Lee S.K., Klostermeyer H. (2014). Effect of the pH of skim milk at heating on milk concentrate viscosity. Int. Dairy J..

[24] IDF (2001). Milk—Determination of Nitrogen Content.

[25] Kadam S.U., Tiwari B.K., O’Donnell C.P. (2015). Effect of ultrasound pre-treatment on the drying kinetics of brown seaweed Ascophyllum nodosum. Ultrason. Sonochem..

[26] Sutariya S.G., Huppertz T., Patel H.A. (2017). Influence of milk pre-heating conditions on casein–whey protein interactions and skim milk concentrate viscosity. Int. Dairy J..

[27] Snoeren T.H.M., Damman A.J., Klok H.J. (1982). The viscosity of skim milk concentrates. Neth. Milk Dairy J..

[28] Rupp L.S., Molitor M.S., Lucey J.A. (2018). Effect of processing methods and protein content of the concentrate on the properties of milk protein concentrate with 80% protein. J. Dairy Sci..

[29] O’Donnell S., Butler F. (2008). Viscosity of reconstituted milk protein concentrate solutions as a function of shear, temperature and concentration. Dev. Chem. Eng. Miner. Process..

[30] Li K., Woo M.W., Patel H., Selomulya C. (2017). Enhancing the stability of protein-polysaccharides emulsions via Maillard reaction for better oil encapsulation in spray-dried powders by pH adjustment. Food Hydrocoll..

[31] Cullen P.J., Duffy A.P., O’Donnell C.P. (2001). In-line consistency monitoring of tomato based products using vibrational process viscometry. J. Food Process. Preserv..

[32] Jaiswal N. (2014). CFD Analysis of Centrifugal Pump: A Review. Int. J. Eng. Res. Appl..

[33] Henning B., Rautenberg J. (2006). Process monitoring using ultrasonic sensor systems. Ultrasonics.

[34] Wallhaußer E., Hussein M.A., Becker T. (2012). Detection methods of fouling in heat exchangers in the food industry. Food Control.

[35] Kazys R., Rekuviene R., Sliteris R., Mazeika L., Zukauskas E. (2015). Ultrasonic technique for monitoring of liquid density variations. Rev. Sci. Instrum..

[36] Bamberger J.A., Greenwood M.S. (2004). Measuring fluid and slurry density and solids concentration non-invasively. Ultrasonics.

[37] Richard B., Toubal M., Le Page J.F., Nassar G., Radziszewski E., Nongaillard B., Debreyne P., Schuck P., Jeantet R., Delaplace G. (2012). Ultrasound tests in a stirred vessel to evaluate the reconstitution ability of dairy powders. Innov. Food Sci. Emerg. Technol..

[38] Hauser M., Amamcharla J.K. (2016). Development of a method to characterize high-protein dairy powders using an ultrasonic flaw detector. J. Dairy Sci..

