# Supplementary material for: Monitoring Viscosity and Total Solids Content of Milk Protein Concentrate Using an Inline Acoustic Flowmeter at Laboratory Scale
Source: Foods. 2020 Sep 17;9(9):1310. doi: 10.3390/foods9091310 (PMC7554928; doi:10.3390/foods9091310)
Supplement: Supplementary file 1 [file foods-09-01310-s001.pdf]

Supplementary data

Supplementary Table 1. Effect of flowrates on the viscosity of MPC85

Flowrate (l/min)	Temperature (45 °C)	Inline viscosity in skid (mPa.s)	Acoustic transmission (%)
2.93 ± 0.02	45.09 ± 0.20	9.54 ± 0.17	80.4 ± 0.19
4.91 ± 0.47	44.5 ± 0.07	9.5 ± 0.13	80.15 ± 0.06
6.34 ± 0.25	45.88 ± 1.11	8.53 ± 0.31	80.38 ± 0.11

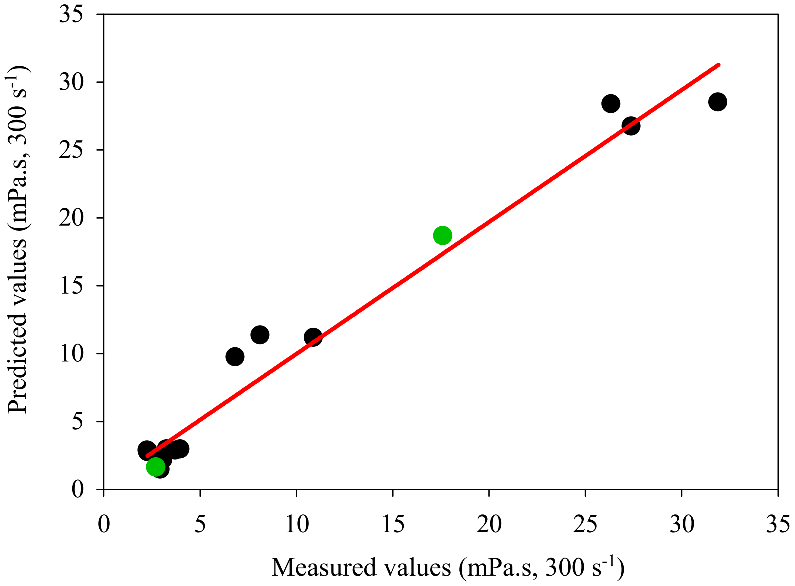

Supplementary Figure 1. Linear correlation between the predicted and measured MPC85 viscosities at laboratory scale (45 °C, 300 s^{-1}): ●: Calibration points, ●: Validation points.

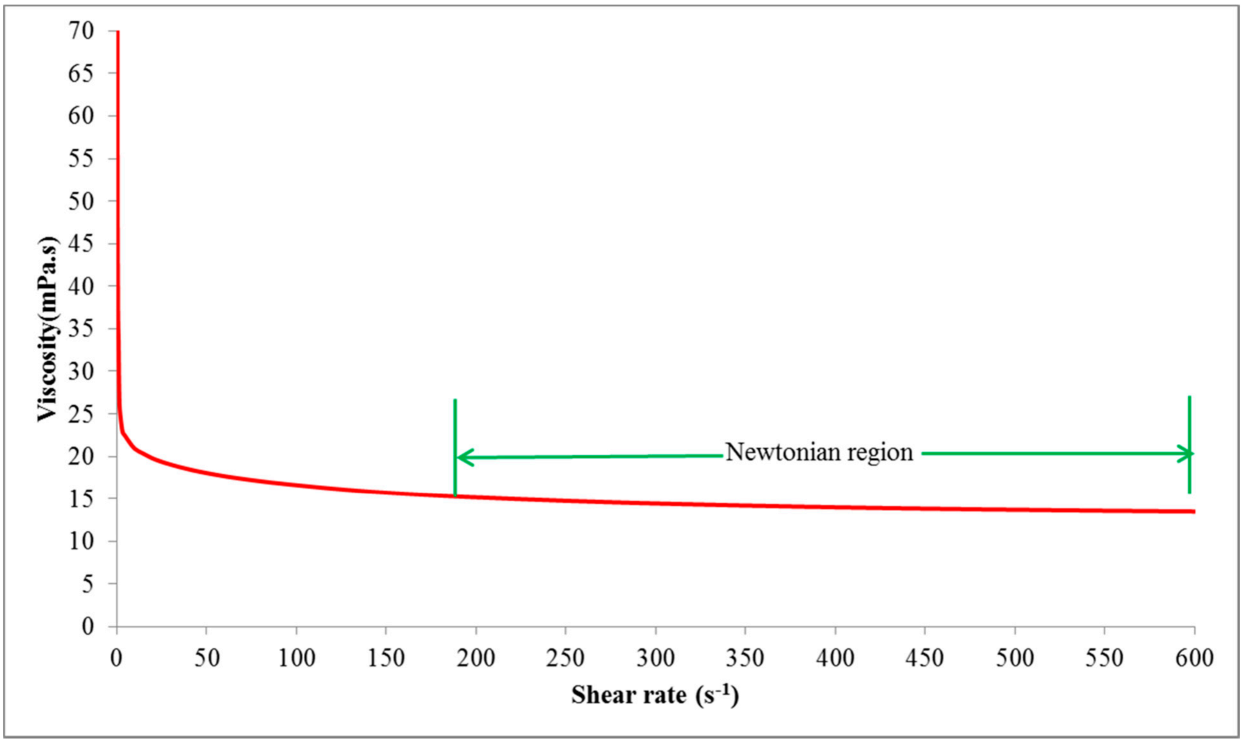

Supplementary Figure 2. Viscosity of MPC85 of 21% TS as a function of shear rate (0-600 s^{-1}) at 45 °C.

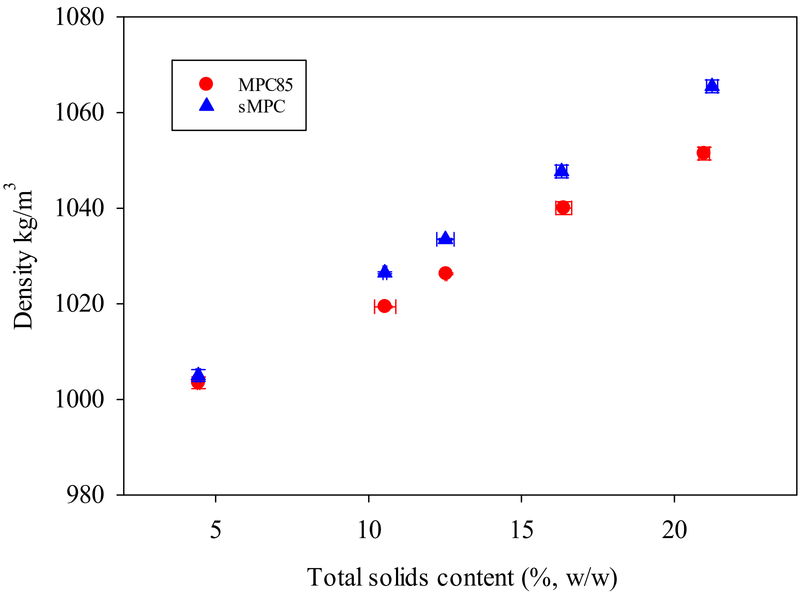

Supplementary Figure 3: Density of MPC85 and sMPC concentrate at 45 °C as a function of TS content (4-21%).
